# Data Storytelling als kritischer Erfolgsfaktor von Data Science

**DOI:** 10.1365/s40702-020-00662-3

**Published:** 2020-10-08

**Authors:** Thomas Neifer, Dennis Lawo, Paul Bossauer, Margarita Esau, Anna-Maria Jerofejev

**Affiliations:** 1 Information Systems, Universität Siegen, Siegen, Deutschland; 2 Wirtschaftswissenschaften, Hochschule Bonn-Rhein-Sieg, Sankt Augustin, Deutschland

**Keywords:** Data Storytelling, Data-Storytelling-Prozess, Datenkompetenz, Datenwissenschaft, Data Storytelling, Data Storytelling Process, Data Literacy, Data Science

## Abstract

Bedingt durch die fortlaufende Digitalisierung und den Big Data-Trend stehen immer mehr Daten zur Verfügung. Daraus resultieren viele Potenziale – gerade für Unternehmen. Die Fähigkeit zur Bewältigung und Auswertung dieser Daten schlägt sich in der Rolle des Data Scientist nieder, welcher aktuell einer der gefragtesten Berufe ist. Allerdings ist die Integration von Daten in Unternehmensstrategie und -kultur eine große Herausforderung. So müssen komplexe Daten und Analyseergebnisse auch nicht datenaffinen Stakeholdern kommuniziert werden. Hier kommt dem Data Storytelling eine entscheidende Rolle zu, denn um mit Daten eine Veränderung hervorrufen zu können, müssen vorerst Verständnis und Motivation für den Sachverhalt zielgruppenspezifisch geschaffen werden. Allerdings handelt es sich bei Data Storytelling noch um ein Nischenthema. Diese Arbeit leitet mithilfe einer systematischen Literaturanalyse die Erfolgsfaktoren von Data Storytelling für eine effektive und effiziente Kommunikation von Daten her, um Data Scientists in Forschung und Praxis bei der Kommunikation der Daten und Ergebnisse zu unterstützen.

## Abstract (en)

Due to the ongoing digitalization and the Big Data trend, an increasing amount of data is available. This results in many potentials—especially for companies. The ability to cope with and evaluate this data is reflected in the role of the data scientist, which is one of the most popular jobs at present. However, challenges arise from the integration of data into corporate strategy and culture. For example, complex data and analysis results must be communicated to stakeholders who are not data-affine. Data storytelling plays a decisive role here, because to use data to initiate change, understanding and motivation for the issue must first be created for every target group. However, data storytelling is still a niche topic. This article uses a systematic literature analysis to derive the success factors of data storytelling for an effective and efficient communication of data to support Data Scientists in research and practice in communicating data and results.

## Einleitung

Data does not go viral. Stories do. (Johnson und Fuoti 2013)

Das Geschichtenerzählen – auch bekannt als Storytelling – ist seit jeher ein wichtiger Bestandteil unserer Gesellschaft. Nicht umsonst nahm die UNESCO das Märchenerzählen, welches nur ein Teil der narrativen Kunst ist, in das Verzeichnis des immateriellen Kulturerbes auf (Deutsche UNESCO-Kommission 2016). Dies wird u. a. auch im aktuellen Diskurs zur Klima- und Coronakrise deutlich, welcher die Relevanz wissenschaftlicher Ergebnisse und deren Kommunikation als Basis für gesellschaftliche und politische Debatten und Entscheidungen aufzeigt (Arnold 2018; Lee und Jahng 2020; Streeck et al. 2020).

Gutes Storytelling ist den Menschen jedoch nicht einfach in die Wiege gelegt. Es ist vielmehr eine Fähigkeit, die erlernt werden muss, um die Aufmerksamkeit seiner Zuhörer gewinnen und halten zu können (Davison 2016). Geschichten helfen, komplexe Sachverhalte zu verstehen und Emotionen zu wecken. Dadurch bleiben diese in Erinnerung und ermöglichen es, die gewonnenen Einsichten zu nutzen und Eindrücke weiterzugeben.

Auch in Unternehmen und der Forschung ist das Erzählen komplexer Sachverhalte essenziell. Durch immer größere Datenmengen und komplexere Analysemethoden eröffnen sich einerseits zwar neue Möglichkeiten zur Erfassung von Sachverhalten, andererseits steigen mit zunehmender Komplexität jedoch ebenfalls die Herausforderungen daran, diese Datenmengen auszuwerten und die Ergebnisse interpretieren, verstehen und kommunizieren zu können (Duan et al. 2019; Hind 2019; Neifer et al. 2020).

Vor diesem Hintergrund gewinnt die Fähigkeit des Data Storytellings, also Daten effektiv und effizient kommunizieren zu können, auch im unternehmerischen Kontext, z. B. der unternehmensinternen Forschung, zentrale Relevanz (Mack et al. 2017; Neifer et al. 2019). Ähnlich verhält es sich mit der Wissenschaft, wie es in der COVID-19-Pandemie, aber auch der Klimawandel-Kommunikation, deutlich wird. Hier zeigt sich ein erhöhter Bedarf an verständlicher und bürgernaher Kommunikation komplexer Datenauswertungen und deren Vorhersagen. In beiden Kontexten kommt hier dem Data Scientist bzw. dem Forschenden – neben seiner besonderen Rolle als Analyst komplexer Zusammenhänge – auch die Aufgabe der Kommunikation an Entscheider zu (Arnold 2018; Christozov et al. 2018; Zhang 2018).

Erfolgreiches Data Storytelling benötigt dazu zwei grundlegende Fähigkeiten, welche, laut Nussbaumer Knaflic (2015), bereits im primären und sekundären Bildungsbereich vermittelt werden. Die Mathematik wird benötigt, um Zahlen zu erfassen, auszuwerten und zu deuten. Die Sprache dient dann dazu, Zahlen in Worte, Worte in Sätze und diese schließlich in Geschichten transformieren zu können.

Trotz der Bedeutung des Data Storytellings für unternehmerische Entscheidungen (Mack et al. 2017) und Wissenschaftskommunikation (Zhang 2018) ist dieses noch ein Nischenthema in der wissenschaftlichen Literatur. Während Storytelling immer wieder als wichtiges Werkzeug aufgeführt wird und sogar bei den Kreativitätstrends des CIO Magazins 2017 gelistet wurde (von Gagern 2017), wurden die Faktoren für den Erfolg des Data Storytellings bisher nicht systematisch untersucht.

Basierend auf einer systematischen Literaturanalyse identifiziert dieser Beitrag acht zentrale Erfolgsfaktoren und leitet daraus einen prozessualen Ansatz für ein effektives und effizientes Data Storytelling zur Implementierung einer nachhaltigen Kultur der datengetriebenen Kommunikation ab, denn: „Data gives you the what, but humans know the why.“ (Bladt und Filbin 2013).

## Data Storytelling

Der Begriff *Data Storytelling* setzt sich aus den Begriffen *Data* und *Storytelling* zusammen. Während *Data* die Zahlen und Zeichen repräsentiert, die zur Entscheidungsfindung analysiert, aufbereitet und kommuniziert werden müssen (Gadatsch und Landrock 2017), wird *Storytelling* als die „Kunst des Geschichtenerzählens“ definiert (Lugmayr et al. 2017).

**Abb. 1:**
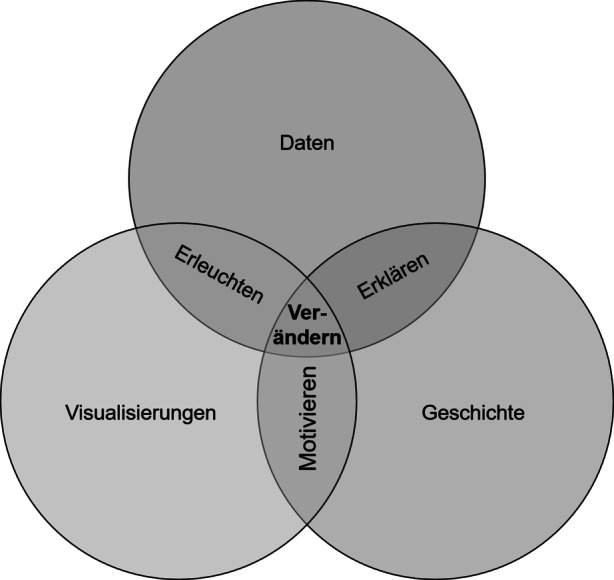
Die drei Schlüsselelemente des Data Storytellings. (Nach Dykes 2016)

Klaus (2019) greift diese Definitionen auf und bezeichnet Data Storytelling als Kommunikationsmethode für „relevante Entwicklungen […] mit dem Ziel, notwendige Entscheidungen zu treffen.“ Weiterhin beschreibt er die Rolle des Erzählers als Dolmetscher der Datenanalyse in eine für jedermann verständliche Sprache (Klaus 2019). Bladt und Filbin (2013) folgen dieser Darlegung und verstehen Data Storytelling als „Vermenschlichung der Daten“. Neben diesen Definitionsversuchen hat sich in der Literatur keine eindeutige Definition des Begriffs durchgesetzt. Basierend auf den verschiedenen Perspektiven und hervorgehobenen Aspekten (Ryan 2016; Vermeulen 2018; Vora 2019; Dykes 2016) des Data Storytellings kann diese jedoch als *Prozess der Informationsaufbereitung und -darstellung von Ergebnissen einer Datenanalyse zur Motivation einer Entscheidung oder Handlung in einer der Zielgruppe entsprechenden Sprache und Visualisierung* verstanden werden. Daraus gehen bereits drei wichtige Elemente des Data Storytellings hervor: Die Daten bzw. Datenanalyse sowie deren zielgruppengerechte Kommunikation mittels sprachlicher Geschichte und verständlichen Visualisierungen. Um eine relevante Geschichte mit Unternehmens- oder Forschungsdaten zu erzählen, ist es notwendig, alle drei Schlüsselelemente des Data Storytellings zu verstehen und zu beachten (vgl. Abb. 1).

Diese Kombination ermöglicht es, den Daten einen Kontext und damit eine Bedeutung im Hinblick auf zukünftiges Handeln zu verleihen. Durch die Visualisierung der Daten können bei den Zuhörern nicht nur Erkenntnisse, sondern auch eine Erleuchtung für neue Blickwinkel und Chancen erzielt werden. Die Verbindung der Geschichte mit Visualisierungen hat ebenfalls Vorteile: Wird eine Erzählung erfolgreich mit visuellen Komponenten unterstützt, kann das Publikum nicht nur unterhalten, sondern auch motiviert werden (Nussbaumer Knaflic 2015). Das Ziel des Data Storytellings, relevante Entscheidungen vorzubereiten und eine Veränderung einzuleiten, wird am besten durch die Verknüpfung aller drei Komponenten verwirklicht (Dykes 2016).

Im Hinblick auf die Geschichte, die Data Story, unterscheidet sich diese hinsichtlich ihrer Struktur nicht von anderen Geschichten. Sie besteht in der Regel aus der Ausgangssituation („in der Vergangenheit“), dem Höhe- und/oder Wendepunkt („dann passiert etwas“) und dem Ende („als Ergebnis“) (Callahan 2016). Nach Ryan (2016) kann zwischen acht Handlungsgerüsten einer Data Story unterschieden werden (vgl. Tab. 1).

**Tab. 1:** Handlungsgerüste von Data Stories. (Nach Ryan 2016)

Plot	Beschreibung
Wandel im Laufe der Zeit	Eine visuelle Geschichte wird durch einen Trend oder eine einfache Metrik erschaffen
Drill-down	Mit dem großen Ganzen beginnen und sich zur Kernaussage vorarbeiten
Zoom-out	Vom Individuellen auf größere Gruppen schließen
Kontrast	Gegenüberstellung zweier Seiten: „Dieses“ oder „Jenes“
Verteilung	Es sollten sowohl die guten als auch die schlechten Seiten betrachtet und die Reichweite der Daten verdeutlicht werden
Übergänge	Dinge, die sich überschneiden oder sich von „weniger als“ zu „mehr als“ ändern
Faktoren	Zwei oder mehr Elemente, deren Effekt sich in Kombination multipliziert
Sonderfälle	Ein wirkungsvolles Mittel ist es, etwas außerhalb der Norm aufzuzeigen

**Abb. 2:**
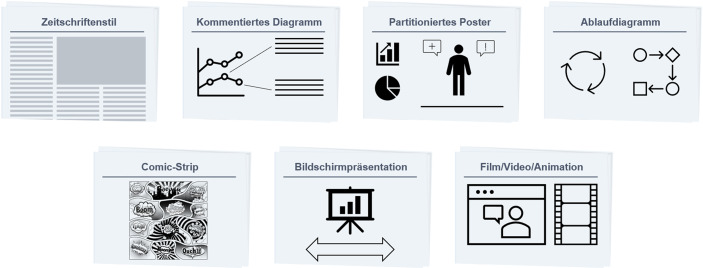
Genres der erzählenden Visualisierung. (Nach Segel und Heer 2010)

Laut Segel und Heer (2010) unterscheiden sich an der Schnittmenge von „Geschichte“ und „Visualisierung“ weiterhin sieben Genres der audiovisuellen Darstellung von Daten (vgl. Abb. 2).

Der Zeitschriftenstil stellt die Einbettung einer einzigen Abbildung in eine Textseite dar, während ein Comic Strip viele Abbildungen in einem linearen Erzählstrang aufweist. Comics bieten besonders im Rahmen von gedruckten Medien den Vorteil, dass eine Data Story mit viel Gestaltungsraum und Ausdruckskraft (Sequenzen mit Bild, Wort und erzählerischen Elementen) versehen werden kann (Bach et al. 2017). Partitionierende Poster, wie sie häufig im wissenschaftlichen Kontext genutzt werden, weisen dagegen meist eine lockere Anordnung der Bilder auf, dies ist insbesondere für leicht verdichtbare Informationen spannend, um unzählige Präsentationsfolien zu vermeiden oder um mehrere Aspekte aufgeteilt und übersichtlich auf einem Poster zu präsentieren. Ein kommentiertes Diagramm, als einfache Form der Bild-Sprach-Beziehung, erzielt Mehrwerte durch die Anreicherung bekannter Diagrammtypen, welche um erklärenden Text ergänzt werden. Ablaufdiagramme unterstützen ein prozessuales Verständnis im Hinblick auf den Zusammenhang zwischen Daten und Events. Im Rahmen von unternehmerischen, aber auch forscherischen Präsentationen, wird statt eines Comic-Strips zumeist auf die Bildschirmpräsentation zurückgegriffen, um eine Data Story aufzubereiten und mit narrativen Elementen zu versehen. Die Darstellung über Filme und Videos wird typischerweise in der Fernseh-Werbung oder Erklärvideos angewandt (Segel und Heer 2010).

Data Storytelling kann dabei einen informierenden, überzeugenden, unterhaltenden, beruhigenden, erklärenden oder erschreckenden Charakter haben (Kelliher und Slaney 2012; Lee et al. 2015; vgl. Tab. 2).

**Tab. 2:** Verschiedene Zwecke von Data Stories

Zweck	Beispiel
Informieren	Anhand aktueller Daten über eine Lage aufklären
Überzeugen	Aufzeigen der Wirksamkeit von Maßnahmen
Unterhalten	Informationen von persönlichem Interesse für die Stakeholder schaffen Aufmerksamkeit
Beruhigen	Daten darstellen, die eine kontinuierliche Entwicklung in turbulenten Zeiten aufzeigen
Erklären	Ein tieferes Verständnis für ein Phänomen anhand detaillierter Fakten schaffen
Erschrecken	Konsequenzen aufzeigen, um zum Handeln zu motivieren

Ojo und Heravi (2018) versuchten anhand von erfolgreichen Data Stories aus der Praxis zu erklären, welchen Mustern und Strukturen erfolgreiche Data Stories folgen (vgl. Tab. 3). Sie analysierten auch, welche Technologien und Werkzeuge dabei zum Einsatz kommen.

**Tab. 3:** Wichtige Muster erfolgreicher Data Stories nach Ojo und Heravi (2018)

Element	Die häufigsten Data Story-Aspekte (in %)
Zweck	Informieren (73) Überzeugen (41) Erklären (39)
Interaktivität	Interaktiv (59) Möglichkeit des Suchens, Filterns und der Auswahl von Daten (27) Statische Graphiken (9)
Darstellung	Kommentierte Graphik (77) Verschiedene/Kombination (18) Video (18) Magazinstil (14) Bilder (11) Slideshow (7)

## Methodik

Um einen Überblick über die Erfolgsfaktoren des Data Storytellings zu bekommen, wurde eine systematische Literaturanalyse, nach den vier Phasen von Rowley und Slack (2004), durchgeführt (vgl. Tab. 4).

**Tab. 4:** Vier Phasen der durchgeführten Literaturstudie

Phase	Vorgehensweise
1. Suche und Evaluierung der Literatur	Durchsuchen von Datenbanken nach Forschungsergebnissen zu Data Storytelling sowie Literatur mit dessen praktischer Anwendung & Qualitätsbewertung nach (Cooper 1988)
2. Filtern und Festhalten relevanter Informationen	Analyse und Zusammenfassung der Literatur nach dem SQ3R-Verfahren (Survey, Question, Read, Recall, Review) zur Identifikation essenzieller Informationen und Literatur (Ridley 2012)
3. Entwicklung eines Konzeptrahmens	Erstellung einer Konzeptmatrix zur Sortierung und Einordnung der identifizierten Literatur (siehe (Webster und Watson 2002))
4. Ausformulieren der Literaturstudie	Herleitung, kritische Begutachtung und Dokumentation einer Theorie aus den zuvor gewonnenen Erkenntnissen

Dazu wurden in der ersten Phase der Literaturstudie Forschungsergebnisse und praxisrelevante Artikel unter Verwendung von Schlagworten, wie z. B. „Data Storytelling“, „Storytelling“ oder „Data Science“ gesucht. Gemäß den Qualitätskriterien von Cooper (1988) und einer Durchsicht der Abstracts hinsichtlich Relevanz wurden diese vorgefiltert, sodass 24 relevante Artikel vorlagen.

Aufbauend auf dieser Auswahl an Literatur, wurde in der zweiten Phase auf das SQ3R-Verfahren (Survey, Question, Read, Recall, Review) zurückgegriffen, um die essenzielle Literatur zu identifizieren (Ridley 2012). Die weitere Auswahl konzentrierte sich auf Forschungsergebnisse, Praktiken oder Anwendungen mit einer neutralen Perspektive und dem Ziel der Generalisierung (Cooper 1988), wodurch aus 24 Quellen die zehn relevantesten Artikel herausgefiltert wurden.

Nachdem die passenden Informationsquellen im Rahmen der ersten beiden Schritte des SQ3R-Verfahrens ausgewählt wurden (Survey, Question), kamen darauffolgend die drei Rs zum Einsatz (Read, Recall, Review), um die relevanten Erfolgsfaktoren zu ermitteln. Dazu wurde eine Konzept-Matrix erstellt (Salipante et al. 1982; Webster und Watson 2002), welche die Ergebnisse der Literaturanalyse in Bezug auf die Erfolgsfaktoren aufzeigt (vgl. Tab. 5). In diesem Schritt wurden ebenfalls ähnliche Erfolgsfaktoren zusammengefasst, sodass insgesamt acht Faktoren identifiziert werden konnten.

**Tab. 5:** Konzept-Matrix

	Erfolgsfaktor							
Quelle	A	B	C	D	E	F	G	H
Nussbaumer Knaflic (2015)	X	X	–	–	X	–	X	–
Klaus (2019)	X	–	X	X	–	–	–	–
Bladt und Filbin (2013)	X	X	–	X	–	–	–	–
Stone (2015)	X	X	–	–	X	X	–	–
Morgan (2016)	–	–	–	–	X	X	–	X
Wellington (2015)	–	X	–	–	X	X	X	–
Samuel (2015)	X	X	X	X	X	–	X	–
Heeg (2015)	X	–	–	–	X	X	X	–
Pyczak (2017)	–	X	–	–	X	X	X	X
Ryan (2016)	X	–	–	X	X	–	–	–

*A* Daten visualisieren, *B* Kernidee in den Fokus stellen, *C* Zum Handeln motivieren, *D* Die richtigen Daten wählen, *E* Komplexität vermeiden, *F* Data Story an Publikum anpassen, *G* Die adäquate Story richtig erzählen, *H* Story gemeinsam erarbeiten

Abschließend wurden die Ergebnisse in Phase vier zu einer Übersicht von Erfolgsfaktoren des Data Storytellings zusammengetragen.

## Die Erfolgsfaktoren von Data Storytelling

**Abb. 3:**
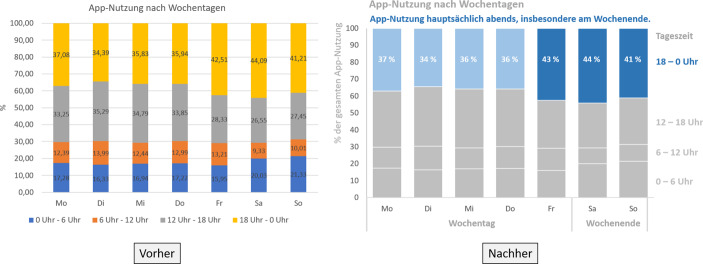
Analyse der App-Nutzung zur Ableitung der relevanten Zielgruppe

Im Folgenden werden die Erfolgsfaktoren (vgl. Tab. 5) anhand ihrer Häufigkeit aufgeführt, näher beschrieben und anhand eines übergreifenden Beispiels eingeordnet. Das Beispiel beschreibt die Ergebnisse einer Analyse der App-Nutzung anhand von Log-Files, welche von einem Forschenden aufbereitet und zur Analyse der Zielgruppe der App präsentiert werden soll (vgl. Abb. 3).

### Komplexität vermeiden

Der am häufigsten genannte Faktor für eine erfolgreiche Data Story ist die Vermeidung von Komplexität. Nussbaumer Knaflic (2015) führt dazu an, dass Komplexität mittels visueller Hilfsmittel reduziert werden kann. Generell sollte darauf geachtet werden, dass eine Data Story leicht lesbar und einfach erzählt ist. Sie untermauert dies damit, dass Zuhörer bei zu kompliziert dargestellten Erklärungen schnell abschalten und sie sich bei einer unnötig komplexen Sprache unwohl fühlen oder sogar verärgert werden (Nussbaumer Knaflic 2015). Auch sorgen komplexe Visualisierungen dafür, dass die Leser eine eigene Geschichte entsprechend ihres Verständnisses entwickeln und dadurch vom gewünschten Zweck abweichen können (Stone 2015). Abbildungen und Texte sollten also nur die wichtigsten Daten und Trends für die eigene Story beinhalten. Anstatt einer Überflutung des Lesers mit allen Daten sollten diese gezielt zur Unterstützung der Aussage genutzt werden (Morgan 2016). Implizit lässt sich bei einigen Autoren bereits die Argumentation zur Findung eines geeigneten Rahmens der Data Story für die jeweilige Zielgruppe erkennen (Heeg 2015; Wellington 2015). Hilfreich ist es in allen Fällen, sich vorher ein interdisziplinäres Feedback einzuholen (Nussbaumer Knaflic 2015). Abb. 3 verdeutlicht, dass eine bessere Farbgebung die relevanten Daten in den Vordergrund stellt und so die Komplexität reduziert werden kann. Das Auge wird direkt auf die wesentliche Information gerichtet und das Verständnis durch den Untertitel gestärkt.

### Daten visualisieren

Die Visualisierung von Daten geht mit der Reduzierung der Komplexität einher und wird von sieben Quellen genannt. Daten sind nach Bladt und Filbin (2013) schwerer in einen Kontext zu bringen. So führen sie anhand eines eigenen Beispiels an, dass die Darstellung von demographischen und geographischen Trends im Rahmen von Regressionstabellen schwer nachvollziehbar ist, insbesondere bei mehreren Merkmalen mit möglichen Interdependenzen. Als sie jedoch auf eine visuelle Präsentation der Daten in Form von Karten zurückgriffen, konnten die Daten schnell durch das Publikum erfasst werden, zur Diskussion anregen und sogar nicht datenaffine Zuschauer befähigen, Trends aufzudecken (Bladt und Filbin 2013). Grafiken sollten dabei jedoch möglichst selbsterklärend sein. Weiterhin ist eine Visualisierung nur als unterstützendes Element anzusehen. Die Geschichte wird nicht durch Bilder ersetzt, sondern zum Höhepunkt geleitet (Klaus 2019). Dies betont auch Heeg (2015) anhand einiger Beispiele aus der Praxis. Er beschreibt, dass die Ableitung von Erkenntnissen aus großen Datenmengen sowie deren Kommunikation v. a. dann nicht erfolgreich verliefen, wenn die Datenvisualisierung nicht als Teil des Data Storytellings wahrgenommen wurde (Heeg 2015). Datenvisualisierungen sollten dementsprechend aktiv in den Data-Storytelling-Prozess integriert werden. Die Visualisierung der Zahlen im Rahmen der Nachher-Darstellung von Abb. 3 verbessert das Data Storytelling durch eine selbsterklärende Aufbereitung und Stützung der Data Story durch eine Fokussierung auf die Haupterkenntnis. Dadurch wird ein Trend hinsichtlich einer vermehrten Nutzung am Wochenende eindeutig erkennbar. Dies ist im Rahmen der Vorher-Abbildung nicht sofort ersichtlich.

### Die Kernidee in den Fokus stellen

Sechs der untersuchten Quellen befürworten es, die Kernidee der Geschichte in den Mittelpunkt zu stellen und die Story darauf aufzubauen. Die Darstellung von Daten sollte immer im Hinblick auf die Adressierung der Frage erfolgen, die mittels der Daten beantwortet werden soll (Pyczak 2017). Unabhängig von den zugrundeliegenden Daten ist es von Relevanz, eine klare Schlüsselbotschaft im Rahmen der endgültigen Data Story zu vermitteln. Dies kann dadurch erreicht werden, dass die Intention eingangs dargestellt wird und im Anschluss linear darauf aufgebaut wird. Hilfreich ist es auch zu reflektieren, wie die Geschichte in einem Satz zusammengefasst werden könnte (Wellington 2015; Samuel 2015). Im Beispiel könnte die Analyse dazu dienen, um Marketing-Maßnahmen zu forcieren, welche hier – aufgrund der hauptsächlich abendlichen Nutzung – über eine Werbung bei einem Streaming-Dienst erfolgen könnte, da die Zielgruppe vorwiegend abends eine Serie ansieht. Anhand der Nachher-Abbildung wird dieser Zusammenhang direkt ersichtlich und kann diskutiert werden. So könnte es weiterhin sinnvoll sein, auch ein Familienpublikum anzusprechen, um die Zielgruppe im Nutzungsbereich von 6–12 Uhr auszubauen.

### Die Story dem Publikum anpassen

Dieser Punkt wurde lediglich von der Hälfte der Quellen explizit angeführt. Allerdings wird die Anpassung der Story an das Publikum im Rahmen dieser Beiträge in den Vordergrund gestellt. Bedingt durch Einheitspräsentationen kommt es so oft dazu, dass die Aufbereitung zwar für die Analysten Sinn ergibt, nicht jedoch für das Publikum. Es ist daher essenziell, sein Publikum zu kennen und die Story entsprechend anzupassen. Durch die sinnvolle Verbindung der Menschen mit der Aussage der Daten, wird so die Überwindung der Barriere zwischen Datenanalysen und Entscheidungsfindung ermöglicht. Hierzu wird empfohlen, jemanden von außerhalb des Vorhabens nach dem Verständnis zu befragen (Heeg 2015; Morgan 2016; Pyczak 2017). Auch hier zeigt die Nachher-Darstellung der Abb. 3 Vorteile gegenüber rohen Zahlen sowie dem Vorher-Diagramm. Durch die übersichtliche und fokussierte Darstellung kann die Marketing-Abteilung den wesentlichen Punkt sofort erkennen und anhand dessen schnell ableiten.

### Die adäquate Story richtig erzählen

Auch die richtige Erzählweise der Data Story wird von 50 % der Quellen genannt. Zur Findung der richtigen Entscheidung ist es wichtig, die unterstützenden Daten zu verstehen. Es kommt hierbei auf die Fähigkeit an, die Zahlen in gute Geschichten zu transformieren. Dabei können nach Pyczak (2017) Erzählstrukturen und Dramaturgie helfen. Für eine richtige Erzählung ist es nach Stone (2015) relevant, sich v. a. auf den Anfang und das Ende eine Story zu fokussieren. Der Beginn einer Geschichte motiviert zur weiteren Aufmerksamkeit, während das Ende die zentralen Botschaften vermittelt, an die man sich erinnert und die weitergetragen werden sollen (Stone 2015). Daten sollten dabei möglichst mit menschlichen Beispielen illustriert werden. Dies sorgt für eine greifbarere Geschichte und macht es einfacher, diese richtig zu interpretieren. Auch die Verwertbarkeit der Botschaften kann dadurch gesteigert werden (Wellington 2015; Samuel 2015). Ein guter Einstieg in die Geschichte des Beispiels könnte die Herleitung der Zielgruppenanalyse über ein amüsantes Bild der App-Nutzung während des Streamings darstellen, da sich damit sicherlich viele identifizieren können.

### Die richtigen Daten wählen

Vier Quellen führen die richtige Auswahl der Daten an. Es geht hierbei primär darum zu verstehen, dass es in Daten nicht zwangsläufig eine Wahrheit gibt und daher der Kontext eine wichtige Rolle einnimmt. Die Daten sowie deren visuelle Darstellung sollten dementsprechend sorgfältig überdacht werden, um eine starke Botschaft präsentieren zu können (Ryan 2016). Auch Bladt und Filbin (2013) argumentieren, dass nur diejenigen Daten einbezogen werden sollten, die sich auf Schlüsselkennzahlen des Unternehmens auswirken und somit auf Daten verzichtet wird, welche die Problemstellung nicht beantworten. Es muss jedoch immer evaluiert werden, ob bei der richtigen Daten- und Visualisierungsauswahl kein falsches Verständnis vermittelt wird (Ryan 2016). In Abb. 3 ist dieser Gedanke durch die Zentrierung auf die relevanten Zahlen hinsichtlich der Identifikation einer Nutzergruppe berücksichtigt worden.

### Story gemeinsam erarbeiten

Von zwei Quellen wird aufgeführt, dass Data Storytelling eine interdisziplinäre Aufgabe darstellt. Dabei kann ein Data-Storytelling-Team aus Datenanalysten, IT-Mitarbeitern, Marketingexperten und Redakteuren bestehen. Datenanalysten extrahieren die Muster und Informationen in den Daten, Experten für Visualisierungen schaffen eine einfache und verständliche Verbildlichung der Daten, Marketingexperten liefern Verständnis für Bedürfnisse und Gedanken des Zielpublikums und Domänenverständnis ermöglicht es, die richtigen Fragen zu beantworten (Morgan 2016). Allerdings verweist Pyczak (2017) darauf, dass eine Person die Story federführend erstellen sollte. Das Team soll Ideen beitragen und die Story verständlich und plausibel gestalten. So sollte die Analyse im Beispiel vor der Anpassung an die Zielgruppe durch interdisziplinäre Abstimmung aus verschiedenen Blickwinkeln diskutiert werden.

### Zum Handeln motivieren

Letztendlich sollen Data Stories das Publikum zum Handeln motivieren. Daher ist es ein relevantes Ziel von Data Storytelling, Neugier zu erwecken, denn dies ist der „Motor der Innovation“ (Klaus 2019). Insgesamt zeigt das Beispiel der Abb. 3, dass Data Storytelling durch die Verbindung von Daten und einer ansprechenden Visualisierung, für eine Erleuchtung über die Hauptnutzungszeit der App für effizientere Marketing-Maßnahmen sorgen kann. Wird die Abbildung mit einer Geschichte kombiniert, so kann sie Motivation schaffen, indem bspw. ein allgemeines Bewusstsein über einen neuen Sachverhalt geschaffen wird. So könnte eine Geschichte bspw. über Streamingdienst-Kunden aufgebaut werden, da diese der wesentlichen Zielgruppe der App entsprechen. Die Daten dienen dabei als Grundlage zur Erklärung der Geschichte und können Aussagen festigen oder weitere Aspekte beleuchten, wie z. B. auch den negativen Trend bei den Nutzern zwischen 6 und 12 Uhr.

Hinsichtlich der Genres ergeben sich für das Beispiel eine Vielzahl von Darstellungsmöglichkeiten. So eignet sich zur Präsentation im Rahmen eines Management-Meetings die Bildschirmpräsentation mit Abb. 3, hierbei könnte je nach Sprachanteil des Redners auch noch das kommentierte Diagramm Anwendung finden. Sollen die Informationen im Anschluss intern in der Marketing-Abteilung verbreitet werden, so wäre ein Comic-Strip denkbar, um Aufmerksamkeit und Verständnis bei den LeserInnen hervorzurufen, ohne dass diese an der Präsentation teilgenommen haben. In einem Bericht könnte der Zeitschriftenstil als Element zur Kommunikation nach außen Verwendung finden.

### Zusammenfassende Betrachtung

Es wird ersichtlich, dass einfach zu verstehende visuelle Darstellungen von Forschung und Praxis als bestes Mittel angesehen werden, um aus reinen Daten eine Story zu gestalten. Interessanterweise wurden drei technische bzw. erzähltechnische Kriterien als essenzielle Faktoren herausgestellt, wohingegen Faktoren, welche sich auf die zu erzählende Geschichte beziehen, nur von jedem zweiten Autor genannt werden.

**Abb. 4:**
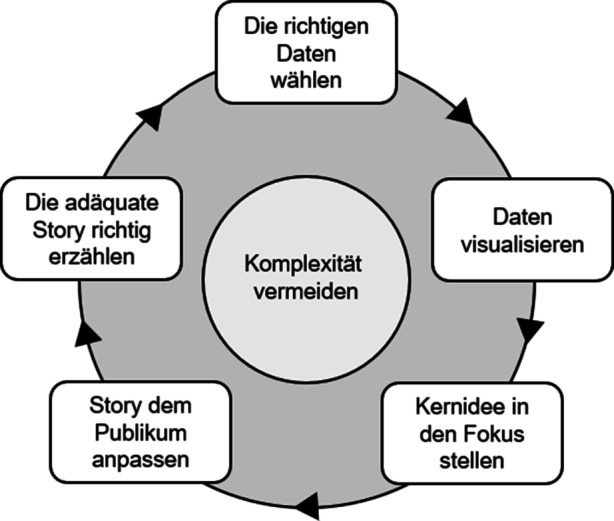
Data-Storytelling-Prozess

Werden die verschiedenen Erfolgsfaktoren in eine zeitlich-logische Abfolge gebracht, so entsteht der von uns empfohlene Data-Storytelling-Prozess. Dieser kann als Leitfaden für die optimale Umsetzung von Data Storytelling angesehen werden (vgl. Abb. 4). Aus einer prozessualen Sicht im Zusammenhang mit Data-Science-Prozessen, wie z. B. dem CRISP-DM-Prozess, kann Data Storytelling als darüber liegender, begleitender Prozess zur effizienten Kommunikation verstanden werden, welcher auch die Phasen der Datenexploration begleitet. Vor dem Hintergrund der geplanten Geschichte und des Zwecks der Story werden die Daten ausgewählt, analysiert und Erkenntnisse gesammelt. Die Formulierung der Geschichte erfolgt über mehrere Iterationen des Prozessmodells. Sie wird dabei durch die Erstellung der zu den Daten der Explorationsphase passenden Abbildungen geprägt (Lee et al. 2015). Schließlich umfasst das Erzählen der Data Story die zielgruppengerechte Verbindung von Geschichte, Visualisierung und Datenanalyseprozess in Kombination mit der geeigneten Wahl des Präsentationsmediums (Ojo und Heravi 2018). Während Data Storytelling den gesamten Prozess begleitet, stellt die eigentliche Kommunikation nicht das Ende des Prozesses dar, sondern motiviert ggf. neue Iterationen der Exploration. So kann z. B. eine ehemals überzeugende Data Story, welche Maßnahmen motiviert hat, in zukünftigen Geschichten der Ausgangspunkt von Evaluation und Information sein.

## Fazit und Ausblick

Der Ausgangspunkt der vorliegenden Arbeit war die Problematik, Daten mithilfe von Storytelling zu kommunizieren. Nicht nur das exponentielle Wachstum von Daten im heutigen Zeitalter, sondern vor allem die Verbindung von Sprache und Mathematik stellen Herausforderungen für das Data Storytelling dar. Um diesen Schwierigkeiten zu begegnen und Daten sowohl in relevante Informationen umzuwandeln als auch das Publikum zum Handeln zu motivieren, wurden Erfolgsfaktoren für ein erfolgreiches Data Storytelling identifiziert. Dies geschah mittels einer systematischen Literaturanalyse.

Das Ergebnis der Auswertung von mehr als 20 Quellen sind acht Erfolgsfaktoren, von denen sechs durch die Häufigkeit ihres Auftretens als besonders relevant angesehen werden. Diese betreffen sowohl den Bereich des Storytellings als auch die Datenanalyse und -visualisierung. Die Faktoren wurden in eine zeitlich-logische Abfolge gebracht und eine Vorgehensempfehlung anhand des so gestalteten Data-Storytelling-Prozesses ausgesprochen. Diese Empfehlung kann von Datenanalysten dazu genutzt werden, erfolgreiche Data Stories zu erzählen und die korrekten Informationen in der richtigen Art und Weise an die Entscheidungsträger zu vermitteln.

Data Storytelling ist jedoch noch immer ein Nischenthema (Mack et al. 2017). Es ist allerdings zu erwarten, dass das Thema aufgrund der steigenden Datenmengen und dem hohen Bedarf an zugeschnittenen Informationen an Wichtigkeit gewinnt (Neifer et al. 2020).
